# IKAP/Elp1 Is Required *In Vivo* for Neurogenesis and Neuronal Survival, but Not for Neural Crest Migration

**DOI:** 10.1371/journal.pone.0032050

**Published:** 2012-02-23

**Authors:** Barbara J. Hunnicutt, Marta Chaverra, Lynn George, Frances Lefcort

**Affiliations:** Department of Cell Biology and Neuroscience, Montana State University, Bozeman, Montana; University of Cincinnatti, United States of America

## Abstract

Familial Dysautonomia (FD; Hereditary Sensory Autonomic Neuropathy; HSAN III) manifests from a failure in development of the peripheral sensory and autonomic nervous systems. The disease results from a point mutation in the *IKBKAP* gene, which encodes the IKAP protein, whose function is still unresolved in the developing nervous system. Since the neurons most severely depleted in the disease derive from the neural crest, and in light of data identifying a role for IKAP in cell motility and migration, it has been suggested that FD results from a disruption in neural crest migration. To determine the function of IKAP during development of the nervous system, we (1) first determined the spatial-temporal pattern of IKAP expression in the developing peripheral nervous system, from the onset of neural crest migration through the period of programmed cell death in the dorsal root ganglia, and (2) using RNAi, reduced expression of IKBKAP mRNA in the neural crest lineage throughout the process of dorsal root ganglia (DRG) development in chick embryos *in ovo*. Here we demonstrate that IKAP is not expressed by neural crest cells and instead is expressed as neurons differentiate both in the CNS and PNS, thus the devastation of the PNS in FD could not be due to disruptions in neural crest motility or migration. In addition, we show that alterations in the levels of IKAP, through both gain and loss of function studies, perturbs neuronal polarity, neuronal differentiation and survival. Thus IKAP plays pleiotropic roles in both the peripheral and central nervous systems.

## Introduction

Familial Dysautonomia (FD) is an autosomal recessive disease whose hallmarks are decreased pain and temperature sensation, a dysfunctional autonomic nervous system marked by cardiac instability, vomiting crises, and renal failure [1]. The stage in neuronal development in which the FD phenotype becomes manifest is not known. An autopsy of a 2-year old FD patient, the youngest age examined to date, showed massive neuronal death including an 80% reduction in neuron number in dorsal root ganglia (DRG) [2] and 90% in FD superior cervical ganglia [3]. Continued depletion of peripheral neurons continues throughout life and results in the progressive degeneration of sensory and sympathetic function.

Although 3 mutations in the *IKBKAP* gene have been identified, the mutation in >99.5% of patients is in a splice acceptor site in intron 20 and results in variable skipping of exon 20, a presumed frameshift, and production of a mutant mRNA [4]–[6]. FD tissues express both wild-type and mutant *IKBKAP* mRNA, although the ratios vary depending upon the tissue type. Of several tissues examined, the central and peripheral nervous systems express the lowest levels of wild type *IKBKAP* mRNA and these are also the tissues most negatively impacted in the disease [5], [6].

The function(s) of the protein encoded by *IKBKAP* gene, which is called IKAP and/or Elp1, is unresolved. Numerous studies indicate that IKAP/Elp1 (from here on, referred to as IKAP) is a subunit of the RNA Pol II Elongator complex [7]–[9]. In addition, a current hypothesis is that IKAP plays a role in cell motility and/or migration [reviewed in 10], [11], with one study providing evidence that it does so by organizing the actin cytoskeleton [12] and other studies indicating a role for IKAP in microtubule organization [13], [14]. Genes downstream of IKAP identified in fibroblasts from FD patients included many genes involved in cell motility [15]. Reductions in IKBKAP mRNA temporarily reduce the migration of cortical neurons and the complexity of their dendritic structure [16]. Given that the cell types that are depleted in FD derive from the neural crest, a highly motile population that migrates to many different target sites deep within the embryo, we sought to investigate whether the IKAP protein is required in the neural crest lineage for its proper migration and/or differentiation.

Neurons in the dorsal root ganglia and sympathetic ganglia differentiate from neural crest cells (NCCs) that delaminate from the trunk neural tube and migrate ventrally: those NCCs that stop next to the dorsal aorta differentiate into the paravertebral chain of sympathetic ganglia while those that stop dorsally, adjacent to the neural tube, give rise to the chain of DRG [17]–[20]. For the PNS to develop properly multiple key steps must occur: neural crest cells must delaminate and migrate to their normal destinations, they must differentiate into the correct cell type at those targets, they must then undergo normal axonogenesis and extend axons that navigate correctly to their required end organs, and a certain percentage must then survive the normal period of programmed cell death. Theoretically disruptions in any of these key events could cause FD.

To determine the steps that go awry to result in the FD phenotype, it is first necessary to identity the stages of PNS development in which IKAP is expressed; no such study has been conducted. We report here that IKAP is not expressed by migrating neural crest cells but instead only starts to be expressed as neural precursors differentiate into neurons, both in the PNS and CNS. Using shRNA, we reduced levels of IKBKAP mRNA *in ovo* and found that alterations in IKBKAP levels affected the genesis, differentiation, polarity and survival of neurons both in the PNS and CNS.

## Materials and Methods

### Construction of shRNA-expressing plasmids

siRNA candidates for IKBKAP were identified using the Whitehead Institute for Biomedical Reseach's website (http://jura.wi.mit.edu/bioc/siRNAext/show_oligo.cgi.). The predicted chicken IKBKAP sequence (NCBI accession number AJ720452) was entered into an analysis program and three siRNAs with negative thermodynamic values were selected for the construction of three hairpin vectors: sequence 5′-AAGTCAGCTGATGTTCCTAGA-3′ (IKP-4), sequence 5′-AACCTTTATCAAGCAAATAGA-3′ (IKP-7), and sequence 5′-AAGTGGCTAGACAGGCTTATG-3′ (IKP-1). These sequences were subjected to BLAST searches using GenBank's nonredundant and EST databases to ensure no off-target gene recognition. Oligonucleotides were annealed and cloned into the pSilencer 1.0-U6 (Ambion, Austin, TX:7208), predigested with ApaI and EcoRI restriction endonucleases. The constructs obtained, psilencer IKP-4.5, psilencer IKP-7.4, and psilencer IKP-1.6 each have their oligonucleotide inserts cloned downstream of the mouse U6 promoter allowing the expression of the short hairpin RNAs. A hairpin plasmid of the same length as the IKBKAP shRNA, 21 nucleotides, and similar % GC content was used as a negative control shRNA. This sequence was also subjected to Blast searches to ensure no gene recognition. To create a second set of siRNA candidates targeted closer to the 5′end of the transcript, an algorithm developed by Maurice Ho at Hung Kong University of Science and Technology, which is based on Reynolds et al 2004 was used. Only the siRNA candidates with scores of 7 or higher were screened for off target sequences as described above, and 2 were selected to make hairpin vectors. One targets the sequence 5′-AGATCTACGTGTACAGATA-3′ (IKP-8) and the other targets 5′-ACAGGCAGTTAATCAGATT-3′ (IKP-3). Oligonucleotides were annealed and cloned into the PBGFPH1 Vector (Gift from Dr. Xiaozhong Alec Wang, Northwestern University). This vector allows for transposition into the genome, giving long-term expression, and co-expression of eGFP by a CMV promoter. The constructs obtained, PBIKP-8 and PBIKP-3 each have their oligonucleotide inserts cloned downstream of the Human H1 promoter allowing the expression of the short hairpin RNAs by RNA polymerase III. The IKP-7 and the Control siRNA described above were also annealed into the PBGFPH1 vector with the same methods.

### CT-IKAP cloning

The C-terminal IKAP construct was generated by amplifying cDNA made from Stage (St.) 28 chicken DRG mRNA and cloning the product into pCS2-His. The C-terminal construct corresponds to bp 3169–4069 in AJ720452. pCS2-His was cut with XhoI and ClaI and then blunt ended with Vent polymerase and checked for correct orientation.

### Injection and electroporation of shRNAs in ovo

For shRNA mediated knockdown experiments, constructs were injected/electroporated into the neural tubes of St.13/14 chick embryos at a concentration of 0.5–2.5 ug/ul. To visualize transfected cells in embryos transfected with IKBKAP shRNAs 7.4, 1.6 and 4.5, the EGFP expression vector pMES was included in the mix at an 8-fold lower concentration. Two of the IKBKAPs generated, 33 and 81 had EGFP encoded in the plasmid and did not require co-injection of pMES; a separate control plasmid was generated when using those 2 shRNAs. For rescue experiments, IKBKAP 7.4 shRNA (1.5 ug/ul) was electroporated in combination with a construct encoding the full length mouse IKBKAP subcloned into pMES (mIKBKAP pMES) at 0.25 ug/ul. As a positive control, embryos were electroporated with mIKBKAP pMES alone (0.25 ug/ul).

### Immunostaining, generation of monoclonal antibodies and in situ hybridization

Immunolabeling and tissue fixation and embedding were carried out as previously described [21]. Fixed tissues were cryosectioned at 16 um. The following antibodies were used: anti-IKAP H302 (Santa Cruz), Tuj-1 (Covance), anti-His (Quiagen) HNK-1, BEN, and Islet-1 (Developmental Hybridomas Bank), anti-cleaved Caspase-3 (Cell Signaling), and all fluorophore-conjugated secondary antibodies (Alexa Fluor 488 nm, 568 nm, and 688 nm) were obtained from Invitrogen (Molecular Probes, Eugene, OR). Monoclonal antibodies were generated against amino acids 1–385 from the partial chicken IKAP sequence (Accession number XP_423369) which corresponds to amino acids 852–1152 in the human IKAP sequence using standard techniques. This region was amplified by PCR and ligated into the pET46 EK/LIC vector. Expressed protein had a 6× His tag and was purified over a Ni column, followed by HPLC. Mice were immunized and monoclonal antibodies generated and screened according to standard techniques (Dr. Elizabeth A. Wayner, Fred Hutchison Cancer Center, Seattle, WA). Monoclonal P1D8 was used for these studies as it gave the strongest signal for immunostaining and western blots. In situ hybridization were conducted according to the same protocol as described in [21]. The probe was made by amplifying the region spanning from nucleotide 3001–3730 in the chick IKBKAP mRNA (Accession number AJ720452) using cDNA from chick E8.5 spinal cord mRNA as a template according to [22]. The PCR-amplified region was then cloned into pCR2.

### RNA extraction and real-time quantitative PCR

Chick embryos (St. 14) were injected/electroporated in the neural tube with shRNAs IKBKAP 1.6+4.5, (target gene; 2.5 ug/ml) or of control shRNA construct (2.5 ug/ml). Embryos were harvested at St. 19–20. A second group of chick embryos (St. 17–18) were injected with 1 ug/ul of either PBIKP 71(target gene), PBIKP 33 (target gene) or PBIKP03 (control). Embryos were harvested at St. 19–20. In all cases, four embryos per construct were harvested. Only electroporated and GFP positive tissues were collected on ice. Tissue was washed twice with RNase free IX PBS and pellets were frozen in liquid nitrogen. RNA was extracted using Trizol, followed by one chloroform extraction and isopropanol precipitation. cDNA was synthesized from RNA (250 ng) using SuperScript III (Invitrogen) according to the manufacturer's instructions. Comparative real–time qRT-PCR [23] was performed in 100-ul tubes using a Rotor gene 6000 (Corbett Research) thermal cycler. PCR was performed using the Rotor –Gene SYBR Green PCR kit (Qiagen). Each cycle consisted of denaturation for 10 seconds at 95 C, annealing for 20 seconds at 58 C, and extension for 25 seconds at 72 C. Primer sequences were as follow: B-actin left: 5′-TGCGCATAAAACAAGACGAG; B-actin right: 5′-GACTGCTGCTGACACCTTCA; IKAP 4 left: 5′-GAGAAGCAAATCCACCAGGA; IKAP 4 right: 5′-CCTGTTTTCCCTCTGATCCA. The amplification efficiencies were determined using the comparative quantitation software supplied by Corbett Research for the rotorgene. The relative quantification ratio was determined using the efficiency corrected calculation model, based on multiple samples [23]. B-actin had a coefficient of variation of 5.2% under all conditions.

### Cell culture

Dorsal root ganglia were dissected from Embryonic day 5–9.5 chick embryos (E5–9.5) and dissociated by incubation in 0.25% trypsin-EDTA (Gibco) for 7 min at 37 C followed by trituration through fire–pulled glass pipettes. The culture media consisted of Neurobasal medium (Invitrogen) supplemented with B27 (1X,Invitrogen), Glutamax (1X,Invitrogen), Hybrimax Antibiotic\Antimicotic (1∶100, Sigma), NGF (10 ng/ml, gift from Dr. Thomas Large). Cells were plated on 8-well Nunc glass chamber slides that were coated with poly-D-lysine (1∶100, Sigma) and laminin 20 ug/ml (Gibco). Approximately equal numbers of cells (52,500) were plated per well. Immediately after plating, cells were transfected with IKBKAP-7.4 shRNA or control shRNA via pn-Fect (Neuromics, PN3375). Several ratios of pnfect∶DNA were tested with the optimum obtained 1.84∶1. The cells were then cultured for approximately 29 h at 37 C, 5.5% C02. After incubation culture cells were fixed and inmunostained as previously described [24]. To determine whether IKBKAP shRNAs altered cell proliferation and/or neuronal differentiation in dissociated DRG cultures, Brdu was added to the cultures and the cells were fixed 24 hrs later (as described in [24]). The number of GFP+/BrdU+ or GFP+/Tuj-1+ cells were quantified for each experiment, and a ratio comparing control vs. IKBKAP shRNAs for each experiment determined. For the BrdU+ experiment, a total of 556 GFP+/control shRNA transfected cells were counted and 357 GFP+/IKBKAP shRNA transfected cells in 3 separate experiment. For determining neuronal differentiation, a total of 2866 GFP+/control shRNA transfected cells and 2913 IKBKAP shRNA transfected cells were counted, over 3 separate experiments.

### Quantification

#### Determination of number of H3+ cells in ventricular zone

Embryos were transfected at St. 13 and fixed at St. 23. Embryos were then cryosectioned and immunolabeled with anti-phosphoH3 antibodies and the number of H3+ cells determined at the ventricular zone on both the transfected and non-transfected sides of the spinal cords for 3 embryos in each condition: Control shRNA, IKBKAP shRNA, or IKPBKAP shRNA+mouse IKBKAP (Origene).

#### Determination of number of DRG neurons in shRNA-transfected embryos

Embryos were injected/electroporated at St. 13 and fixed at St. 24/25. Three shRNA-transfected embryos were analyzed for each of two different IKBKAP shRNAs and the control shRNA. Slides were labeled with Tuj1 or Ben, two neural markers, and the percentage of GFP+/Tuj-1+ or GFP+/Ben+ cells determined in DRG sections from each embryo.

#### Determination of number of cleaved Caspase 3+ cells

Embryos were injected/electroporated as above, incubated until St. 25, fixed, sectioned and immunolabeled with an antibody to cleaved caspase 3. The number of cleaved caspase 3+ cells in the DRG of embryos was determined on both the transfected and non-transfected sides of the embryos and a ratio generated to insure that cell death was a direct result of the shRNAs and not due to potential non-specific effects of electroporation. The number of cleaved-caspase 3+ cells was also determined in the transfected half of the spinal cords in IKBKAP shRNA (n = 3 embryos; 59 sections) and control shRNA transfected embryos (n = 2 embryos; 46 sections).

#### Determination of fate of CT-IKAP-His-tagged cells in the PNS

Embryos were transfected with the CT-IKAP-His pCS2 or a control pCS2 plasmid encoding His and a myc tag at St. 13 and fixed at St. 21. Embryos were then cryosectioned and stained with HNK-1 and/or Tuj-1 antibodies to identify the cell types and with anti-His or anti-Myc tags to identify transfected cells.

#### Branching of ventral root fibers

Chicken embryos St. 11–14 (average 13/14) were electroporated with IKBKAP shRNAs or control shRNA. Electroporated embryos were harvested at St. 23–26, fixed in 4% PFA for 1–2 hrs, cryosectioned and immunolabeled. The number of GFP+ branches emerging from the ventral root or floor plate from the electroporated side of the spinal cord, were quantified. A branch was considered a fiber that deviated from the fascicle of axons emerging laterally from the ventral root, or as individual fibers protruding ventrally from the floor plate. Branches emanating from below the notochord were not included in the analysis.

#### Statistics

Statistical significance for each data set (with the exception of the number of pH3+ cells in Figure 4) was determined by unpaired Student's t-test. Statistical significance for the pH3+ data set was determined by ANOVA.

## Results

### IKBKAP expression begins as neural precursors differentiate

**Figure 1:**
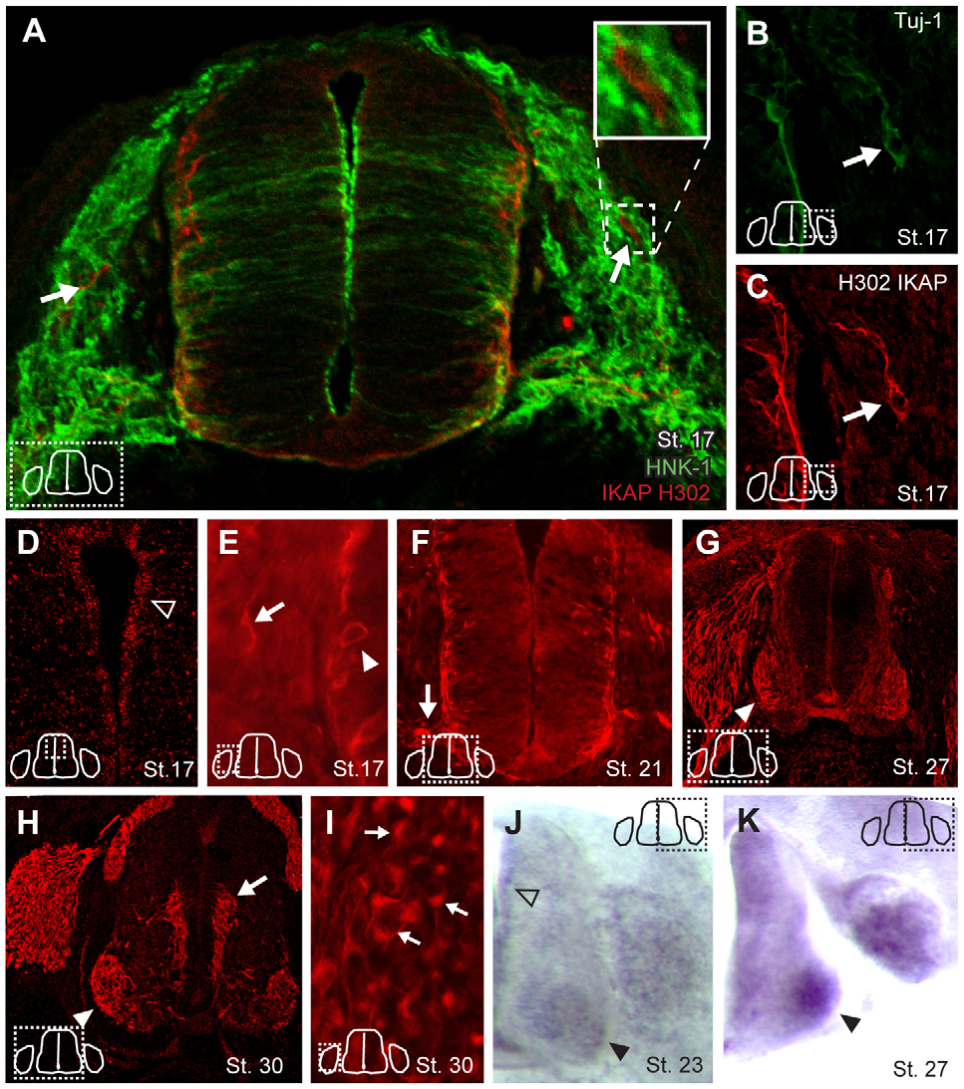
IKAP is expressed in PNS and CNS neurons, but not in neural crest cells. Chick embryo sections were immunolabeled with commercial anti-IKAP antibody (Santa Cruz H302; red, A–I and with either HNK-1 (A; to label neural crest cells, green) or Tuj-1 (to label neurons; B). IKAP protein (red, A–I) is not expressed in HNK+ neural crest cells (green, A), but is expressed in nascent neurons in the DRG anlagen (A–C, E, arrow) and neural tube (B–C, E; arrowhead), and its expression is maintained through the period of programmed cell death (G–I). IKAP is also expressed in progenitor cells in the ventricular zone (D,J open arrowheads) and in motor neurons (arrowhead) and axons (arrow) (F–H,J,K). IKAP is also strongly expressed on the pre-ganglionic Column of Terni cells (arrow, H). In situ hybridization for IKBKAP confirms the protein expression pattern (J,K).

FD devastates the sensory neurons in the dorsal root ganglia and yet no careful temporal examination of *IKBKAP* expression in the developing DRG or PNS has been conducted. We analyzed both protein and mRNA expression for IKAP/*IKBKAP* during the period of neural crest migration and DRG formation and differentiation: St. 15/Embryonic Day (E) 2.5 – E9 (Figure 1). In the trunk, the neural crest delaminate from the neural tube over a period of 24 hrs from ca Hamburger and Hamilton (HH) St. 12 to HH St. 20 [18], [19], [25], [26]. DRG development then proceeds over the next few days with the onset of programmed cell death beginning around E4.5 and lasting until ca E12 [27], [28].

Importantly, all reagents tested failed to detect any IKAP protein or mRNA in migrating neural crest cells (Fig. 1A–C). Instead, in both the spinal cord and DRG anlagen, IKAP protein and mRNA were first expressed in nascent neurons, beginning at St. 17 (Fig. 1A–C, E, arrows). IKAP expression increases as sensory neurons mature in the DRG (Fig. 1G, H, and I) and is expressed during the period of programmed cell death (Fig. 1 G–I, K). These results are consistent with the demonstration of *IKBKAP* mRNA expression in the E21 rat DRG [29] and with expression we and others have observed in the embryonic mouse DRG [30; Lefcort and George, data not shown]. In addition, our data demonstrate pronounced expression of IKAP/*IKBKAP* in the developing spinal cord, particularly in the ventral horn motor columns (Fig. 1G, H, J, K solid arrow head) and their axons (Fig. 1F, arrow), in interneurons along the lateral margins of the cord (Fig. 1C, E, arrowhead, F)and in the ventricular zone (VZ, Fig. 1D,J arrowhead outline). The expression in the ventricular zone is coincident temporally with mitogenesis that occurs here. The protein and mRNA expression patterns complemented each other well, with the major difference being the absence of IKBKAP mRNA in axons. At higher magnification it was apparent that the IKAP protein was strongly cytoplasmic, primarily perinuclear with strong aggregation at the axon hillock region wherein the Golgi apparatus resides (Fig. 1 E, I, arrows) and robustly expressed within both central and peripheral axons. IKAP is also strongly expressed in the pre-ganglionic Column of Terni neurons (Figure 1H, arrow), which are the pre-ganglionic neurons that innervate sympathetic neurons in the paravertebral chain of ganglia.

**Figure 2:**
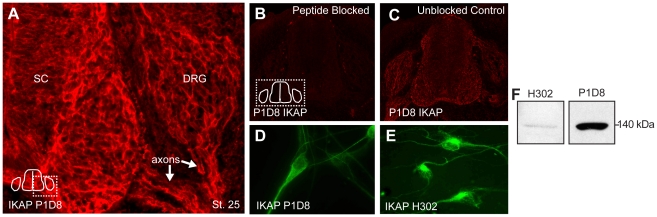
Confirmation of IKAP expression with an anti-chick IKAP antibody. (A) Generation of a monoclonal antibody to chicken IKAP, P1D8, confirms the expression pattern obtained with anti-human IKAP commercial antibody (H302). Both antibodies demonstrate that the majority of IKAP is in the cytoplasm, primarily perinuclear, and in axons (A, C–E) both *in vivo* (A, C) and in DRG *in vitro* (D,E). Incubation with the peptide immunogen used to generate P1D8 completely blocks labeling (compare B to C). Both IKAP antibodies label a band of approximately 140 kDa in lysates of chick embryo spinal cord (F).

Since this immunostaining series was conducted with a commercial anti-Human IKAP antibody (Santa Cruz H302), we generated monoclonal antibodies to chicken IKAP and tested whether they confirmed the expression pattern obtained with the commercial antibody, which they did (Figure 2). Preabsorption with the immunogen used to generate the anti-chick IKAP antibody blocked the immunolabeling (compare Figure 2B to 2C). Both antibodies (P1D8 and H302) strongly label axons, and the perinuclear and axon hillock regions of DRG neurons *in vivo* and cultured *in vitro* (Fig. 2D,E). At this level of resolution it is difficult to exclude that there may be some nuclear expression of IKAP, but certainly the majority of IKAP protein is localized in the cytoplasm. Western blots on lysates made from chick E9 spinal cord (Figure 2F) show that both antibodies react with a band of approximately 140 kDa. This is slightly smaller than the human and mouse IKAP (150 kDa), however, no full length cDNA encoding the chicken IKAP has yet been described so the molecular weight for chicken IKAP is currently unknown.

To summarize, IKAP expression in the PNS begins at St. 17 as neural precursor cells differentiate into neurons; it is not expressed in neural crest cells. IKAP also commences expression in the spinal cord as motor neurons and interneurons differentiate. In addition, IKAP is expressed on the apical surfaces of neuroepithelial cells in the ventricular zone.

**Figure 3:**
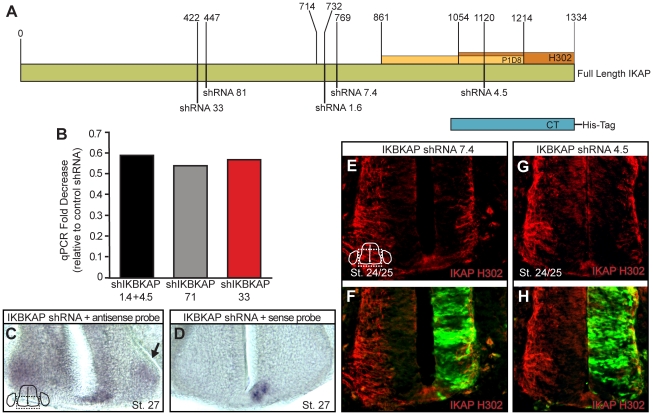
IKBKAP shRNAs reduce levels of IKAP protein and IKBKAP mRNA *in ovo*. (A) Schematic combines the known partial chicken IKBKAP sequence with the human IKBKAP sequence and indicates the regions of chicken IKBKAP to which shRNAs were designed and the regions used as the immunogens for generation of both antibodies (P1D8 and Santa Cruz H301). The cytoplasmic terminus of the chicken IKAP was used for the CT-IKAP His construct overexpression experiments. QPCR indicated that approximately 50% of the IKBKAP mRNA was reduced following a 24 hr transfection *in ovo* with the IKBKAP shRNAs, when compared to control levels of IKBKAP (B). In situ hybridization following IKBKAP shRNAs transfection *in ovo* also showed reduced levels of IKBKAP mRNA (C) in the transfected half of the spinal cord (arrow); sense probe (D). Transfection *in ovo* with IKBKAP shRNAs reduced levels of IKAP protein (E–H) on the transfected side only (compare the control half to the EGFP+ half of the spinal cord).

To determine the function of *IKBKAP* in the developing DRG, we generated shRNAs to 5 regions of the chicken *IKBKAP* gene (Figure 3A) and two control shRNAs (Methods) and transfected these constructs into the developing neural tube *in ovo*. We injected/electroporated shRNAs beginning at St. 12, prior to neural crest emigration and incubated embryos for 1–4 days. All 5 shRNA constructs generated very comparable results and were used interchangeably throughout the studies. Evidence of the ability of the shRNAs to reduce *IKBKAP* mRNA expression is demonstrated in Fig. 3B–D and to reduce IKAP protein expression in Figure 3 E–H by comparing the intensity of staining on the control (left) vs. the electroporated/transfected sides (right) of the spinal cord.

### IKBKAP is required for normal CNS development

Although our study was aimed at investigating the function of *IKBKAP* in the PNS, in order to transfect the neural crest, the neural tube lumen is injected/electroporated before neural crest delamination, along its anterior/posterior axis thereby transfecting the neural tube unilaterally. Given that we had detected robust expression of *IKBKAP* mRNA in the neural tube, we asked whether reductions in *IKBKAP* mRNA might also perturb spinal cord development. Recent studies show that reductions in *IKBKAP* mRNA in cortical neuroblasts delayed their migration [16] and that gene expression is altered in oligodendrocytes in the cerebrum of FD patients [31]. In addition, CNS deficits such as seizures, abnormal EEGs, poor motor coordination and altered brainstem function have been reported in FD patients [32]–[35].

**Figure 4:**
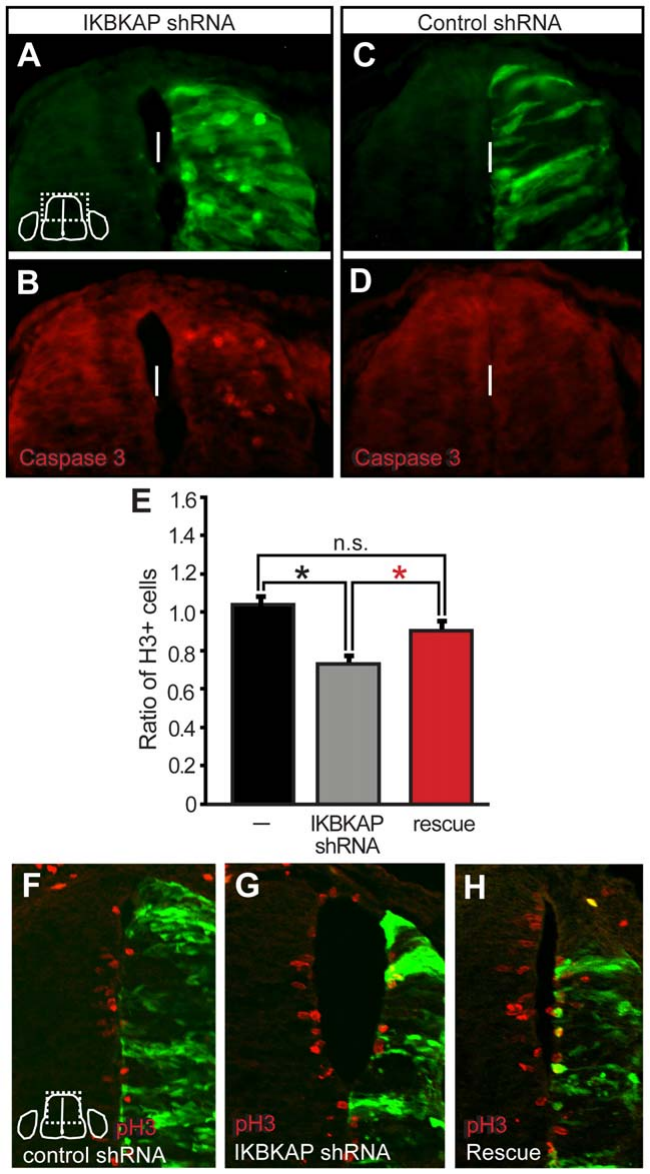
Reductions in IKAP decreases proliferation of progenitors and increases cell death in the developing neural tube. Embryos were transfected/electroporated with IKBKAP shRNAs (A,B) or control shRNAs (C,D) at St. 13 and fixed at St. 23 (ca 48 hrs). Reductions in IKAP increase the death of CNS progenitors as indicated by cleaved-caspase 3+ (A–D; vertical line indicates the midline). (E–H) The number of pH3+ cells was determined in the ventricular zone on the transfected and control sides of each embryo and a ratio for each embryo obtained. The graph depicts those ratios from embryos transfected with either control shRNA or IKBKAP shRNA or IKBKAP shRNA and a mouse IKBKAP cDNA. There are significantly fewer pH3+ cells in IKBKAP shRNA transfected embryos (n = 4 embryos, 3300 cells counted) than in control shRNA transfected embryos (n = 3 embryos, 4,331 cells counted; p = 0.004), an effect that is rescued (red bar) with co-transfection with a plasmid encoding full length mouse IKBKAP (E; n = 3 embryos, 2153 cells counted; p = 0.005 by ANOVA). There is no significant difference between the number of pH3+ cells in the rescued embryos vs. the control shRNA transfected embryos (p = 0.103).

**Figure 5:**
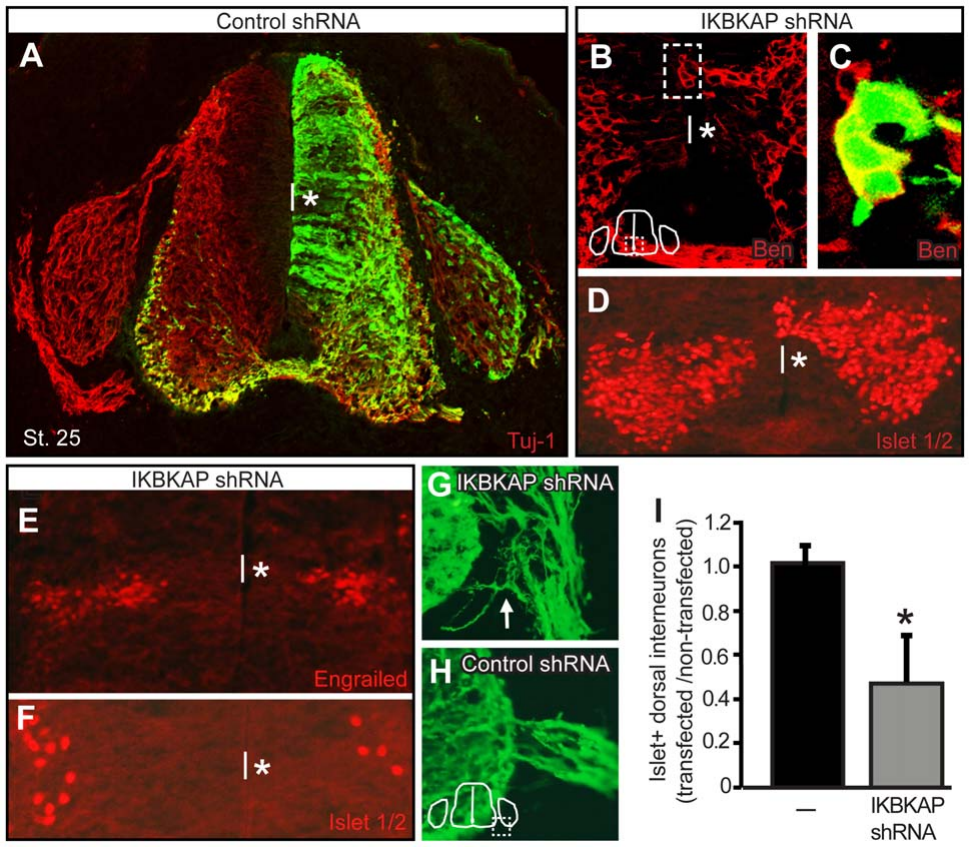
Reductions in IKAP alter neural tube development and induce ectopic branching of motor axons. All transfected cells (control shRNA transfected embryos in A, H, I; IKBKAP shRNA in B–G) are EGFP+; right side of spinal cord is transfected in all embryos (asterisk). (A) control shRNA transfected spinal cord develops normally in contrast to (B–D) IKBKAP shRNA transfected spinal cords in which cells at the ventricular zone (VZ) often evaginate into the neural tube lumen rather than migrate laterally away from the VZ as in (A). IKBKAP shRNA-transfected evaginated cells typically express neural markers such as Ben (C: box in B contains the Ben+ evaginated neurons in the VZ that are EGFP+ and magnified in C) and Islet1/2 (D). Spinal interneuron populations are reduced in IKBKAP shRNA-transfected spinal cords including Engrailed+ cells (E) and the dorsal population of Islet1+ cells – compare the left, non-transfected side of the spinal cords to the right, transfected sides (*asterisk) of the spinal cord. (F, I) The ratio of islet 1+ dorsal interneurons on the transfected vs. non-transfected sides of the spinal cords was determined in multiple sections from 2 control shRNA transfected embryos (n = 263 Islet+ cells for the control shRNA transfected side vs. n = 252 Islet+ cells for the non-transfected side) and in 4 IKBKAP shRNA transfected embryos (n = 356 Islet+ cells for the transfected side of the IKBKAP shRNA spinal cord vs. n = 658 Islet+ cells for the non-transfected sides of the IKBKAP shRNA transfected embryos); p = 0.006. (G, H) IKBKAP shRNA transfected motor axons branch abnormally (arrow in G) as they exit the neural tube in the ventral root (compare H to I).; vertical line indicates spinal cord midline.

The transfected halves of IKBKAP shRNA-spinal cords were typically narrower than the non-transfected side (Fig. 3 E,F). This may be due to either an increase in cell death in IKBKAP shRNA-transfected cells and/or a decrease in the number of mitotically-active progenitors. We quantified the number of cleaved-caspase 3+ cells in the spinal cord and found while control shRNA transfected spinal cord halves contained 0.9+1.43 cleaved caspase3+ cells, IKBKAP shRNA-transfected spinal cord halves had 4.4+1.59 cleaved caspase3+ cells, a 4-fold increase in cell death (p = 0.000; Fig. 4A–D). To investigate changes in mitotic activity of progenitors at the ventricular zone, we quantified the number of phosphorylated histone H3+ progenitors (Fig. 4E–H). We found that reductions in IKAP reduced the number of cycling progenitors, an effect that was rescued by co-transfection *in ovo* with a plasmid encoding full length mouse *IKBKAP*. Another common effect of IKAP reduction was evagination of transfected ventricular zone cells into the neural tube lumen (Figure 5B–D): interestingly, these evaginated transfected cells expressed neural markers such as Ben (Fig. 5B, C) and islet 1/2 (Figure 5D). Reductions in *IKBKAP* mRNA also manifested in a reduction in various spinal cord interneuron populations including the Engrailed and dorsal islet1+ interneuron subpopulations (Fig. 5 E,F,I).

Lastly, reductions in IKAP caused aberrant branching from spinal motor axons in the ventral root (arrow, Figure 5 G,H). We measured approximately a 2-fold increase in the number of branches extending from IKBKAP shRNA-transfected motor axons in the ventral root (mean number of branches = 0.41+0.056 SEM, n = 161 sections counted; p = 0.002) than in control-shRNA-transfected motor axons (mean number of branches = 0.19+0.042 SEM, n = 116 sections counted). Thus *IKBKAP* is required for normal genesis and differentiation of CNS neurons.

### IKBKAP regulates neuronal differentiation and survival in dorsal root ganglia

**Figure 6:**
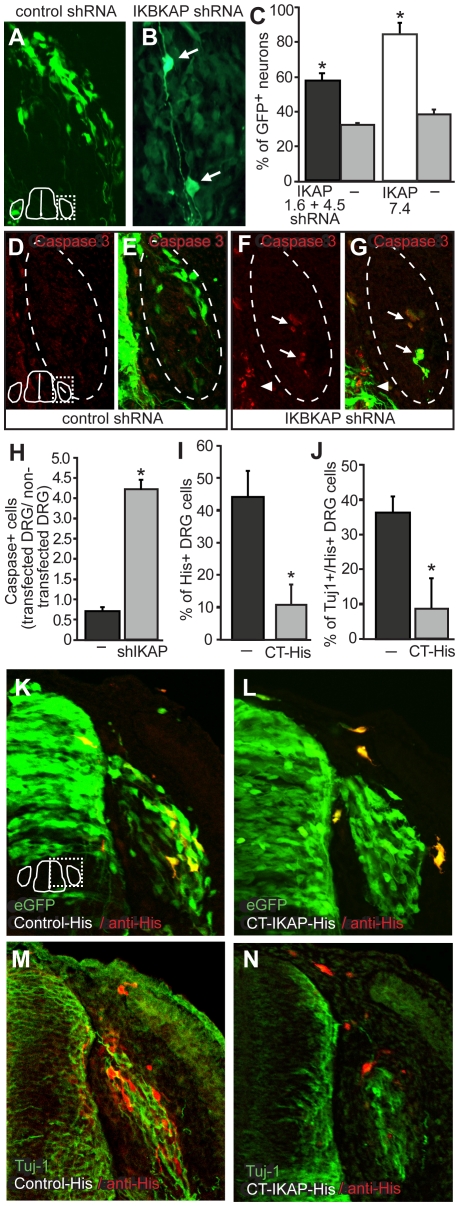
IKAP regulates neuronal differentiation in the DRG. Reduction in IKAP leads to increased numbers of neurons in the immature DRG (A–C). Embryos at St. 12 were transfected with either control shRNAs or IKBKAP shRNAs and analyzed at St 24/25. Embryos were sectioned, and immunolabled with the neuronal markers Tuj-1 or Ben and the percentage of GFP+ neurons determined. Significantly more IKBKAP shRNA transfected DRG precursor cells differentiated into neurons (arrows in B; IKBKAP shRNA 7.4; n = 3 embryos; p = 0.002; IKBKAP shRNA 1.6 & 4.5, n = 3 embryos; p = 0.001) than Control shRNA transfected DRG precursor cells (n = 5 embryos). (D–H) IKBKAP shRNA-transfected DRG precursor cells (n = 3 embryos; 252 cleaved-Caspase 3+ cells counted) were also more likely to die by apoptosis (compare D & E to F & G) than control shRNA-electroporated cells (n = 3 embryos; 117 cleaved caspase-3+ cells counted). (H) The number of cleaved Caspase 3+ cells was quantified in DRG on both the transfected side of the embryo and the non-transfected side of the embryo and a ratio determined. Significantly more cleaved-Caspase 3+ cells were present in the transfected DRG of IKBKAP shRNA transfected embryos than in the transfected DRG in embryos transfected with control shRNAs; p = 0.006. (I–N). Over-expression of the c-terminus of IKAP prevents neural crest cells from coalescing with the DRG (I, K, L; p = 0.004) but the few that do join, tend not to differentiate into neurons (p = 0.007; J, M, N). Embryos were transfected with either a construct driving expression of the c-terminus of chicken IKAP with a His tag (CT-IKAP-His; L,N) or a control, His-tagged construct (K, M) and analyzed at St. 21. The location and neuronal identity of transfected cells was determined in 3 embryos for each treatment; Control His-plasmid: n = 343 transfected cells counted; CT-IKAP-His: n = 278 transfected cells counted. Statistical analysis by Student t-test.

Control and IKBKAP-shRNA-transfected neural crest cells migrated normally and stopped appropriately in the incipient DRG or sympathetic ganglion. We determined the fate of shRNA-transfected cells in the DRG at E4.5/HHSt. 25. At this age, the DRG is considered “immature” in that 30% of its cells are mitotically-active progenitors and neural differentiation is just getting underway [36]. While in control shRNA transfected DRG, GFP+ cells were distributed throughout the ganglion and morphologically appeared as both neurons and non-neurons (Figure 6A), *IKBKAP* shRNA-transfected cells were sparse in number and tended to have the morphology of mature differentiated neurons (Figure 6B). Quantification of GFP+/Tuj-1+ cells supported this observation and demonstrated that silencing of *IKBKAP* with any of the 3 different shRNA constructs tested, promoted premature neuronal differentiation of transfected precursor cells in the DRG (Figure 6C). We next asked whether there was a bias in terms of the type of sensory neuron the *IKBKAP* shRNA transfected cells differentiated into, i.e. TrkA, B or C+ cells, since they correspond to distinct functional modalities. The distribution of Trk expression was comparable to the normal distribution of Trk co-expression at that age, with roughly half of the neurons being TrkC+, one third TrkB+ and one third TrkA+ (data not shown; e.g. see 21 for description of Trk co-expression patterns).

We were struck by the sparse number of transfected cells present in the DRG and tested whether enhanced cell death might occur in *IKBKAP* shRNA transfected DRG. The normal period of programmed cell death in the DRG begins at E4.5 [27], but the numbers are quite low initially and gradually increase until E12. While transfection with control shRNA did not cause an increase in cell death in the DRG, transfection with each of three different *IKBKAP* shRNAs led to a significant increase in the number of cleaved Caspase-3+ cells in the DRG (Fig. 6D–G, H). Since the major hallmark of FD is a 90% reduction in neuronal numbers in the DRG, our data suggest that the deficit could be due to both a decrease in the absolute number of neurons initially generated in the DRG due to premature depletion of the precursor pool, in addition to their enhanced and premature death.

### Ectopic expression of the IKAP C-terminus disrupts sensory neurogenesis

The c-terminus of IKAP contains TPR-like motifs which are found in proteins that mediate mitosis and neurogenesis [37]. The IKAP c-terminus has been shown to bind JNK and is sufficient for JNK activation [38]. Given that it is the c-terminal half of IKAP which would be absent in FD cells should any truncated protein be made, we sought to investigate its function in the developing DRG. Embryo neural tubes were electroporated *in ovo* with a construct encoding the c-terminus of chicken IKAP (CT-IKAP-His; see schematic in Figure 3A) and incubated for 24 hrs. CT-IKAP transfected neural crest cells delaminated and migrated, however a significantly lower percentage than control-transfected neural crest cells colonized the DRG proper and instead were located laterally or dorsally to the DRG (Fig. 6 I, K–N). Of the few cells that did coalesce with cells within the DRG, a significantly lower percentage than control-transfected cells differentiated into neurons (Fig. 6 J, M,N).

### A role for IKAP in neuronal polarity, branching and cytoskeleton organization

Reductions in *IKBKAP* mRNA have been shown to decrease expression of several genes involved in cell motility and exocytosis, to disrupt microtubule organization and decrease branching of cortical neurons [12], [13], [15], [16]. We therefore examined whether *IKBKAP* reductions altered the morphology, polarity and/or cytoskeleton of neurons in the DRG.

**Figure 7:**
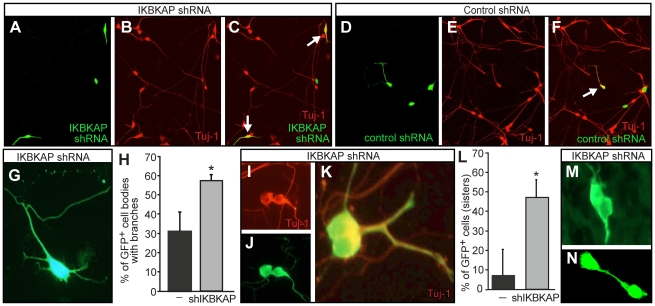
Reductions in IKAP increase neuronal branching and perturb cell separation. DRG were dissociated, transfected with IKBKAP shRNA or control scrambled shRNA and cultured for 24 hrs. (A–F). Reductions in IKAP increased the number of neurons in cultures from immature (E5) DRG. (G–L) Reductions in IKBKAP increased branching from neuronal somata/cell bodies (G,H, p = 0.013) and resulted in incomplete cytokinesis (I–L) or separation of nascent sister neurons *in vitro* (p = 0.01; G–L; 4 experiments, n = 135 control shRNA transfected neurons; n = 116 IKBKAP shRNA transfected neurons) and *in vivo* (M, N; St. 25).

Since we could not transfect mature DRG neurons *in ovo* directly, we instead dissected DRG from E5–E6 embryos and transfected dissociated DRG cells *in vitro* and cultured cells for 24 hrs. At this age about 30% of the cells in the DRG are cycling neural precursors [24], [39]. We first quantified neuronal numbers (Tuj1+/GFP+) to determine whether increased neuronal numbers were also generated from transfected cells *in vitro* as they were *in ovo*. There was a 70% increase in neurons in wells transfected with IKBKAP shRNAs vs. control shRNA - transfected wells (Fig. 7A–F; n = 3 experiments; p = 0.001) consistent with our findings *in ovo*. We also measured potential effects of the *IKBKAP* shRNAs on cell proliferation *in vitro* by including a pulse of BrdU and found a 40% decrease in the number of transfected BrdU+/IKBKAP shRNA transfected cells compared to control shRNA transfected cells (n = 3 experiments; p = 0.022). This result supports our *in ovo* finding that reductions in IKAP led to premature differentiation of neural precursors.

Reductions in IKAP altered the morphology of DRG neurons: it promoted a significant increase in branching from the cell body (Fig. 7 G,H), and increased the generation of two conjoined neurons, which stayed interconnected to various degrees, via a cytoplasmic bridge and/or by incomplete cytokinesis (Fig. 7 I,J), or directly juxtaposed [Fig. 7K] or extensively interconnected via filopodia. There were significantly more pairs of such conjoined neuronal cells in the IKBKAP-shRNA transfected cultures than in control shRNA transfected wells (Fig. 7L). Given that the percentage of neurons was low in each culture (approximately 10%) and hence each individual neuron was easily detectable and at low density, and the fact that the conjoined cells typically had mirror symmetric morphologies, these conjoined neurons are most likely sister cells and daughters of a shared transfected mother-precursor cell. Tightly juxtaposed (Fig. 7M) or interconnected (Fig. 7N) DRG neuronal cells were also commonly observed *in ovo* in *IKBKA*P shRNA transfected embryos. These conjoined neurons were frequently heavily interconnected, if not through filopodial branches, than directly at the junction of the two cells as if cytokinesis was incomplete. These data point to an effect of *IKBKAP* on the cytoskeleton and suggests that it is required for the normal transition from cell cycle completion to the differentiation of daughter cells.

## Discussion

It is well established that the clinical manifestations of FD result from a depletion of neurons in the PNS, yet the steps that go awry to produce this deficit have not been determined. The data presented here are the first *in vivo* studies that identify the stages of PNS development that require IKAP/Elp1. For example, the FD neuronal deficit in the dorsal root ganglia could result from problems with neural crest cell induction, delamination, migration, and/or differentiation. Our study indicates that neural crest cells are induced, delaminate and migrate normally when transfected with IKBKAP shRNAs. This finding is supported by the fact that IKAP is not expressed in neural crest cells; thus the postulate that FD results from disruptions in neural crest motility and/or migration are not supported by investigation of IKAP's function and expression *in vivo* reported here. Similarly, our data demonstrate that the disease phenotype does not result from a failure in neuronal differentiation, since peripheral neurons differentiate from IKBKAP-shRNA-transfected neural precursors. Rather, our data provide evidence that the deficit in neuronal numbers that marks FD may result from two causes: (1]) peripheral precursor cells precociously differentiating into neurons and (2) neurons prematurely dying. These findings may shed insight on the pathological basis for the FD phenotype.

Using RNAi to investigate the mechanisms underlying the PNS phenotype in FD is an appropriate and useful strategy given the fact that FD tissues express varying levels of both wild type IKBKAP mRNA and truncated mRNA [6] and hence FD cells are not truly “null” for IKAP protein. Thus our *in vivo* model is more analogous to the human disease than a knock-out mouse model would be, even a conditional knock-out. This is further supported by the fact that the *IKBKAP* knock-out mouse is embryonic lethal, dying by E9 [30], [40; Lefcort and Tessarollo, unpublished].

A major finding reported here is that neurons expressing reduced levels of IKAP died prematurely in the DRG; the mechanisms mediating that death need to be elucidated. IKAP-depletion has been shown to increase levels of pro-apoptotic genes such as Bax in colon cancer-derived cells and to alter levels of a number of pro-apoptotic genes in FD stem cells [41], [42] and may explain the developmental delay and early death observed in IKAP null mice [40]. Yeast two hybrid studies have identified binding of IKAP/Elp1 to BAT-3, an apoptosis regulatory gene [43]. Our finding of premature differentiation of precursors followed by precocious death is reminiscent of the premature differentiation and subsequent death of DRG neuronal precursors in the neurotrophin-3 (NT-3) knock out mouse [44].

Our shRNA data suggest that IKAP plays a role in maintaining neural precursor proliferation and preventing their precocious differentiation into neurons. Decreasing IKBKAP mRNA levels both *in vivo* and *in vitro* led to an increase in neuronal differentiation, yet IKAP levels only become detectable as neural precursors differentiate. Furthermore, overexpressing the cyto-domain of IKAP reduced neuronal differentiation. Without more structure/function studies we can not distinguish whether the ectopically expressed truncated c-terminal IKAP protein acted as a dominant negative or as a gain of function. Johansen et al [2008] showed that overexpression of the N-terminal half of IKAP, either in the presence or absence of the endogenous full length IKAP caused a defect in cerebellar neuronal migration *in vitro*. Holmberg et al [2002] showed that overexpression of the c-terminal half of IKAP was sufficient to cause activation of the critical signaling enzyme, JNK. Interestingly, Johansen et al also showed that in IKAP-depleted cells *in vitro*, ectopic expression of the N-terminal half of IKAP could not rescue the effects of IKAP depletion. Furthermore, in the IKAP null mouse, Chen et al. [2009] found that overexpression of the N-terminal half of IKAP could not rescue the grave early embryonic lethality induced by total loss of IKAP in mouse.nor could generation of a mouse in which only exon 20 was deleted [40]. These last experiments indicate that key functions for IKAP must reside in its C-terminal half, hence the rationale for our experiment. Furthermore, these data would suggest that the level of IKAP is critical and that small alterations in the amount of IKAP in a PNS precursor cell can shift the behavior of that cell between proliferation vs. differentiation. Unfortunately there are no robust markers of chick DRG precursor cells, but our data would suggest that IKAP must begin to be expressed as cells exit the cell cycle and acts as a “break” to prevent precocious differentiation. Decreases in Elp1 in *Arabidopsis thaliana* has been shown to cause a decrease in proliferation [45], and FD cells have been demonstrated to be in cell cycle arrest, an effect released by phophatidylserine [46]. IKAP-dependent genes include such cell cycle mediators as p57kip and thymidilate synthetase [15]. Since the cytoskeleton and cell polarity proteins are so integral to both cell division and cell motility, its not surprising, given the accruing evidence for a role for IKAP in cell polarity, that we found alterations in cell proliferation and cell polarity with disruptions in IKBKAP. As published in previous studies, we too found fewer paxilin-positive focal adhesions with IKAP reduction [12] (data not shown). Since paxillin has been shown to mediate the localization of both Crk and syntaxin-2 in the midbody during cytokinesis, IKAP-regulated paxillin might be required for normal cytokinesis [47].

Our data suggest that IKAP/Elp1 participates in the organization or remodeling of the cytoskeleton during cytokinesis and in branching from somata and axons. ShRNA knock-down of IKBKAP mRNA in fibroblasts also induced an increase in branching from cell bodies [13]. Since DRG neurons do not normally extend dendrites, perhaps a normal function for IKAP/Elp1 is to suppress branching in the PNS.

RNAi for IKAP in neuroblastoma cells and in fibroblasts caused the disorganization of microtubules [13]. IKAP has also been shown to bind to Filamin A, a protein that organizes the actin cytoskeleton and interacts with cell adhesion pathways [12]. Interestingly, X-Linked Periventricular Heterotopia is due to mutations in Filamin A and is marked by an aggregation of neurons along the surface of the ventricular zone. In our IKBKAP shRNA treated spinal cords, we saw an aberrant evagination of cells into the lumen of the neural tube which went on to express neuronal markers – an observation also seen in embryos in which the neural tubes overexpress cadherins [48]–[50]. IKAP/Elp1 is expressed in the ventricular zone both in the brain [16] and in the spinal cord. Thus the integration of signaling between IKAP, filamin A and cadherins may be essential for orchestrating the reorganization of cytoskeleton during the transition from cell cycle withdrawal to neuronal differentiation in the CNS ventricular zone.

There has been a recent surge in interest in interactions between IKAP/Elp1 and Elp3 with evidence that Elp3 mediates histone H3 acetylation, and most recently, acetylation of α-tubulin [16], [51]. Acetylation of microtubules leads to stabilization and recruitment of motor proteins that enable transport of proteins and other cellular components [52]. Work in c.elegans showed that decreasing Elp1 reduced the velocity of dense core vesicle movement along axons by regulating microtubule acetylation [14]. In our study and also in [13] we did not find any reductions in tubulin acetylation in the PNS [data not shown], which perhaps points to differences in the function of IKAP/Elp1 in the PNS vs. CNS, and/or to differences in Elp1 and Elp3 interactions in the PNS vs. the CNS.

This study is the first to directly address whether FD results from a problem in cell motility, a hypothesis which is currently popular [e.g. 11]. Our data do not support a role for IKAP/Elp1 in the motility and migration of neural crest cells, a result not surprising given that we show here that IKAP only becomes detectable as neurons begin to differentiate in the DRG. Our data suggest that IKAP coordinates the intracellular machinery required for the transition from neural precursors to neurons and in the differentiation and survival of neurons in the PNS. Further elucidation of the molecular interactions and intracellular pathways in which IKAP participates will be required to fully understand the cellular mechanisms that result in FD.
